# Supplementary material for: The Early Evolution of Oral Poliovirus Vaccine Is Shaped by Strong Positive Selection and Tight Transmission Bottlenecks
Source: Cell Host Microbe. 2021 Jan 13;29(1):32–43.e4. doi: 10.1016/j.chom.2020.10.011 (PMC7815045; doi:10.1016/j.chom.2020.10.011)
Supplement: Document S1. Figures S1–S5 and Tables S1–S5 [file mmc1.pdf]

Cell Host & Microbe, Volume *29*

Supplemental Information

The Early Evolution of Oral Poliovirus Vaccine Is Shaped by Strong Positive Selection and Tight Transmission Bottlenecks

Andrew L. Valesano, Mami Taniuchi, William J. Fitzsimmons, Md Ohedul Islam, Tahmina Ahmed, Khalequ Zaman, Rashidul Haque, Wesley Wong, Michael Famulare, and Adam S. Lauring

Supplemental Information

The early evolution of oral poliovirus vaccine is shaped by strong positive selection and tight transmission bottlenecks

Andrew L. Valesano, Mami Taniuchi, William J. Fitzsimmons, Md Ohedul Islam, Tahmina Ahmed, Khalequ Zaman, Rashidul Haque, Wesley Wong, Michael Famulare, Adam S. Lauring

Table S1: Genome amplification primers used in this study, related to STAR Methods.

Name	Sequence
PanSabin_Seg1_Fwd	5'-CCCGYAACTTAGAMGCA-3'
PanSabin_Seg1_Rev	5'-CTGACACAAAMCCMAGSATG-3'
PanSabin_Seg2_Fwd	5'-TCTGCCCRGTKGATTAYCTC-3'
PanSabin_Seg2_Rev	5'-TCAGTRAATTTYTTCAACCAACT-3'
PanSabin_Seg3_Fwd	5'-GTMAATGATCACAACCC-3'
PanSabin_Seg3_Rev	5'-GTTGGAAAGTTGTACATTAG-3'
PanSabin_Seg4_Fwd	5'-TGTCCTTTAGTGTGTGG-3'
PanSabin_Seg4_Rev	5'-CCCAATCCAATTCGACTG-3'

Table S2: Validation of within-host variant identification by sequencing mock populations, related to STAR Methods, Figure 1, and Figure 2.

		(1) replicate, 4.5 x 10^4 copies/μL[a]			(2) replicates, 9 x 10^3 copies/μL[a]		
Coverage	Frequency	Sensitivity	Specificity	FP[b]	Sensitivity	Specificity	FP[b]
200x	10%	1	1	0	1	1	0
	5%	1	1	0	0.94	1	0
	2%	0.6	1	0	0.49	1	0
	1%	0.17	1	0	0.06	1	0
500x	10%	1	1	0	1	1	0
	5%	1	1	0	1	1	0
	2%	0.91	1	0	0.74	1	0
	1%	0.54	1	0	0.4	1	0
1000x	10%	1	0.9999	1	1	1	0
	5%	1	1	0	1	1	0
	2%	1	1	0	0.97	0.9999	1
	1%	0.91	1	0	0.69	1	0

		(2) replicates, 9 x 10^2 copies/μL[a]			(1) replicate, 9 x 10^2 copies/μL[a]		
Coverage	Frequency	Sensitivity	Specificity	FP[b]	Sensitivity	Specificity	FP[b]
200x	10%	0.89	1	0	1	0.9995	7
	5%	0.83	1	0	0.80	0.9994	9
	2%	0.4	1	0	0.46	0.9990	14
	1%	0	1	0	0.31	0.9997	4
500x	10%	0.97	1	0	1	0.9991	13
	5%	0.97	1	0	0.97	0.9985	21
	2%	0.49	1	0	0.51	0.9984	23
	1%	0.03	1	0	0.51	0.9992	12
1000x	10%	1	1	0	1	0.9987	19
	5%	1	1	0	0.97	0.9974	37
	2%	0.63	1	0	0.91	0.9985	22
	1%	0.03	0.9999	2	0.03	0.9989	16

[a] Copies/μL is 1000-fold lower than copies/gram of stool.
[b] Number of identified false positives.

Table S3: Gene-wise estimates of dN/dS ratio, related to Figure 2.

Gene	Omega (dN/dS)
VP4	0.26326
VP2	0.05951
VP3	0.44309
VP1	1.20709
2A	0.25478
2B	0.00010
2C	0.05137
3A	0.09244
3B	0.00010
3C	0.10622
3D	0.04747

Table S4: Samples from transmission pairs used in bottleneck analysis, related to Figure 5.

Donor ID[a]	Recipient ID[a]	Donor Vaccination Date[b]	Donor Sample Date[c]	Recipient Sample Date[d]	Time Difference (days)
115	10115	2016-01-26	2016-02-02	2016-02-01	1
171	10171	2016-01-25	2016-01-31	2016-01-31	0
702	20702	2016-01-25	2016-02-08	2016-02-08	0
927	10927	2016-01-28	2016-02-24	2016-02-17	7

[a] Anonymous IDs per individual.
[b] Date of mOPV2 administration in trial vaccination campaign.
[c] Date of sample collection from mOPV2 recipient used in the bottleneck analysis.
[b] Date of sample collection from the household contact used in the bottleneck analysis. For each recipient, this is the first longitudinal sample positive for OPV2 by RT-PCR.

Table S5: Transmission bottleneck estimates for two models, related to Figure 5.

Pair ID	Presence-absence model estimate[a]	Beta-binomial model estimate[a]
115	1 (1 – 2)	1 (1 – 3)
171	2 (2 – 4)	2 (2 – 7)
702	2 (2 – 2)	2 (2 – 3)
927	2 (2 – 5)	2 (2 – 4)

[a] 95% confidence interval shown in parentheses.

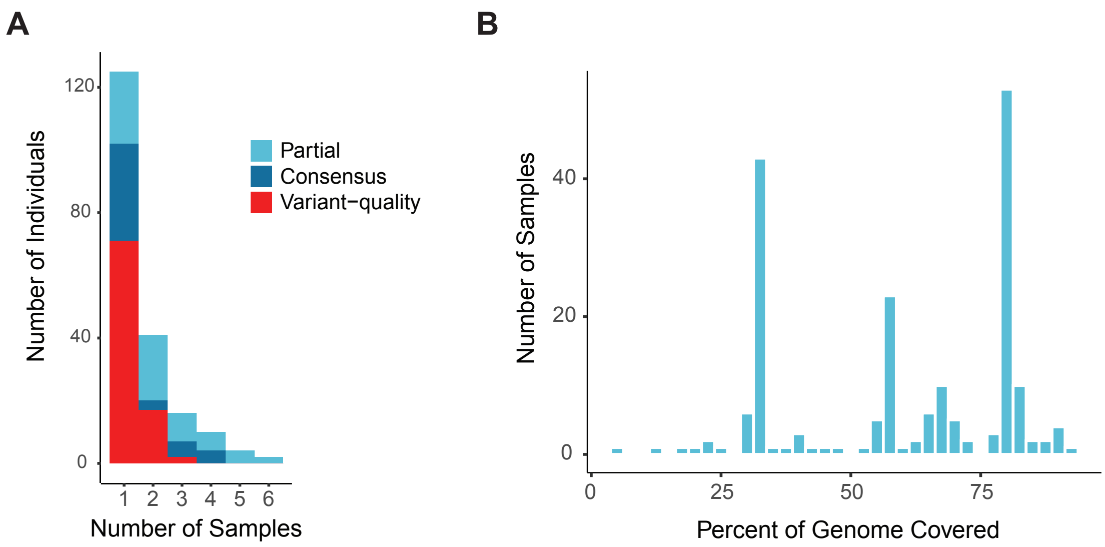

Figure S1. Sequencing Coverage, Related to Figure 1

(A) Overlapping bar chart of the number of individuals (y-axis) by the number of samples sequenced from a given individual (x-axis). Colors represent the genome coverage groups shown in Figure 1. (B) Composition of the partial genome samples. Number of samples (y-axis) by the percent of the genome covered above a 10x threshold (x-axis).

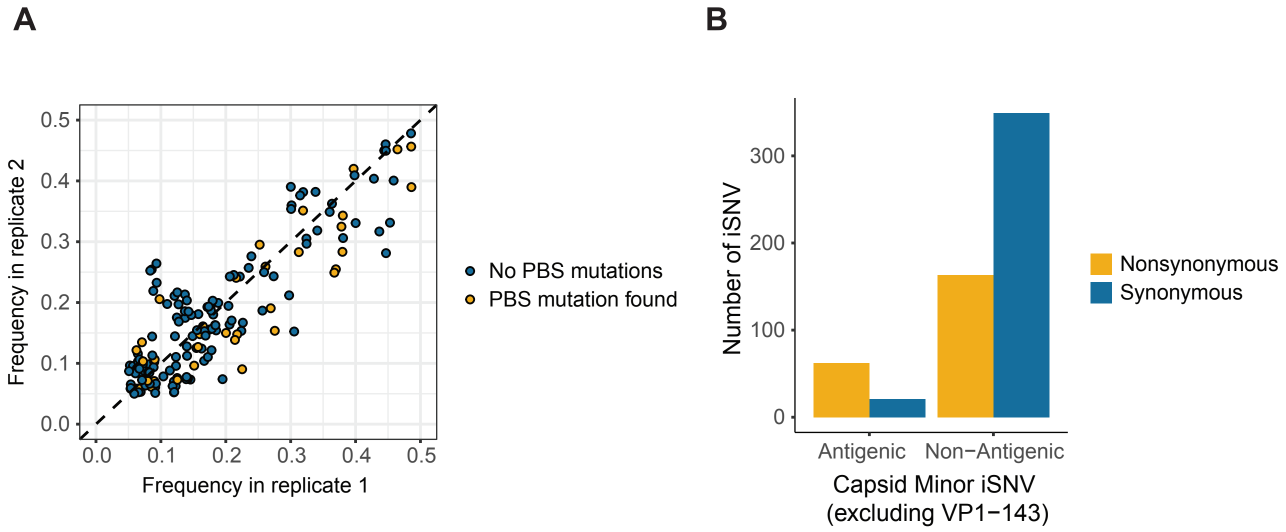

Figure S2. Minority iSNV, Related to Figure 2

(A) Concordance of iSNV frequency measurements across 11 samples sequenced in duplicate. Frequency of iSNV in replicate 2 (y-axis) is shown by the frequency of an iSNV in replicate 1 (x-axis), with colors showing iSNV on amplicon(s) with or without mutations in primer binding sites. (B) Histogram of minor iSNV in the capsid region by antigenic status, excluding VP1-143. Nonsynonymous iSNV are shown in yellow, and synonymous iSNV in dark blue.

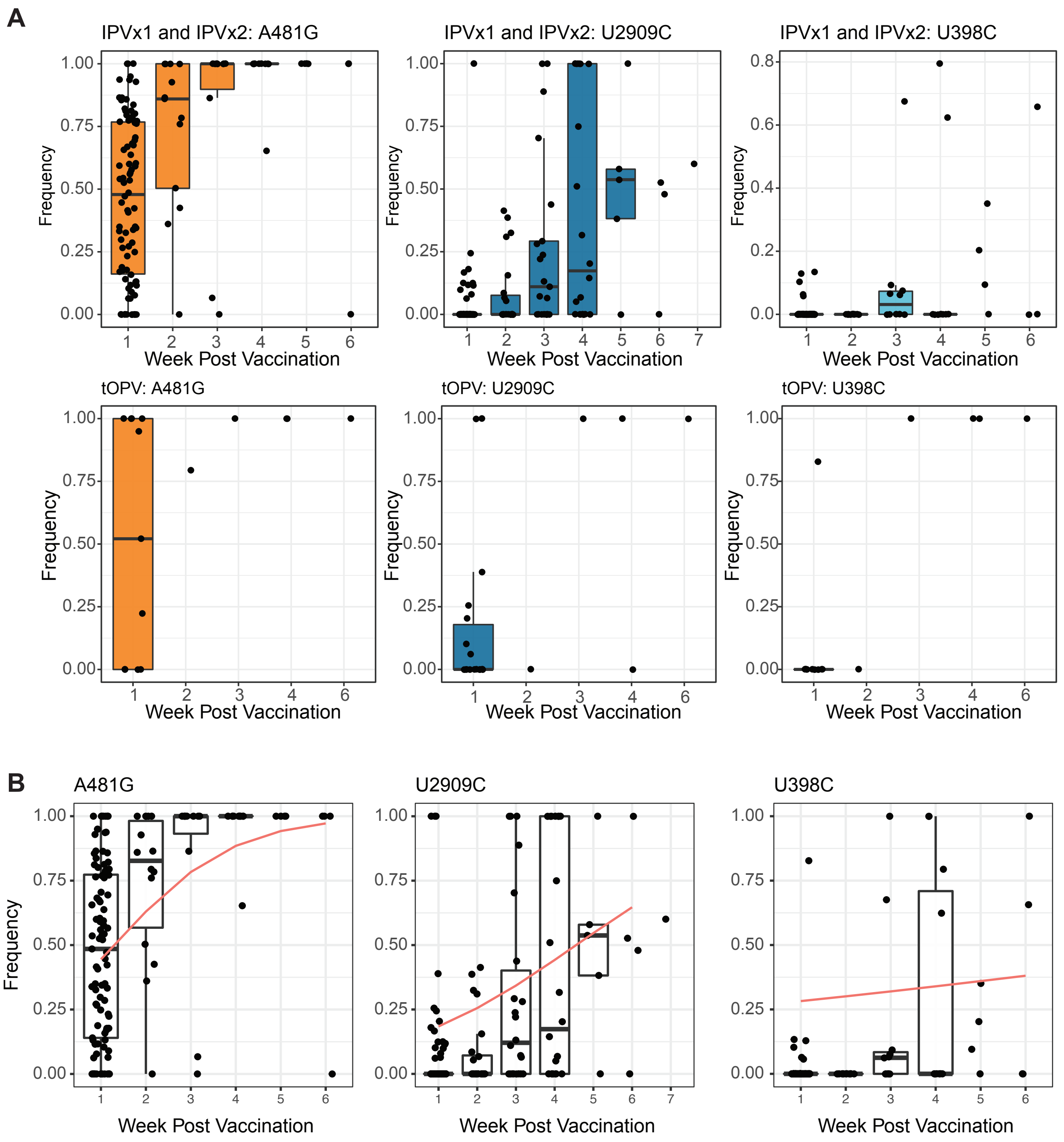

Figure S3. Gatekeeper Mutations, Related to Figure 3

(A) Frequency of A481G, VP1-143X, and U398C by time from vaccination across arms of the vaccine trial. Samples from IPV arms are shown on the top, and samples from tOPV arms are shown on the bottom. Each point represents one sample, and boxplots are shown for weeks with five or more data points. Boxplots represent the median and 25th and 75th percentiles, with whiskers extending to the most extreme point within the range of the median ± 1.5 times the interquartile range. (B) Frequency of A481G, VP1-143X, and U398C by time from vaccination across with the beta regression model fits for each mutation (red lines). The underlying data are the same as in Figure 3A. Each point represents one sample, and boxplots are shown for weeks with five or more data points. Boxplots represent the median and 25th and 75th percentiles, with whiskers extending to the most extreme point within the range of the median ± 1.5 times the interquartile range.

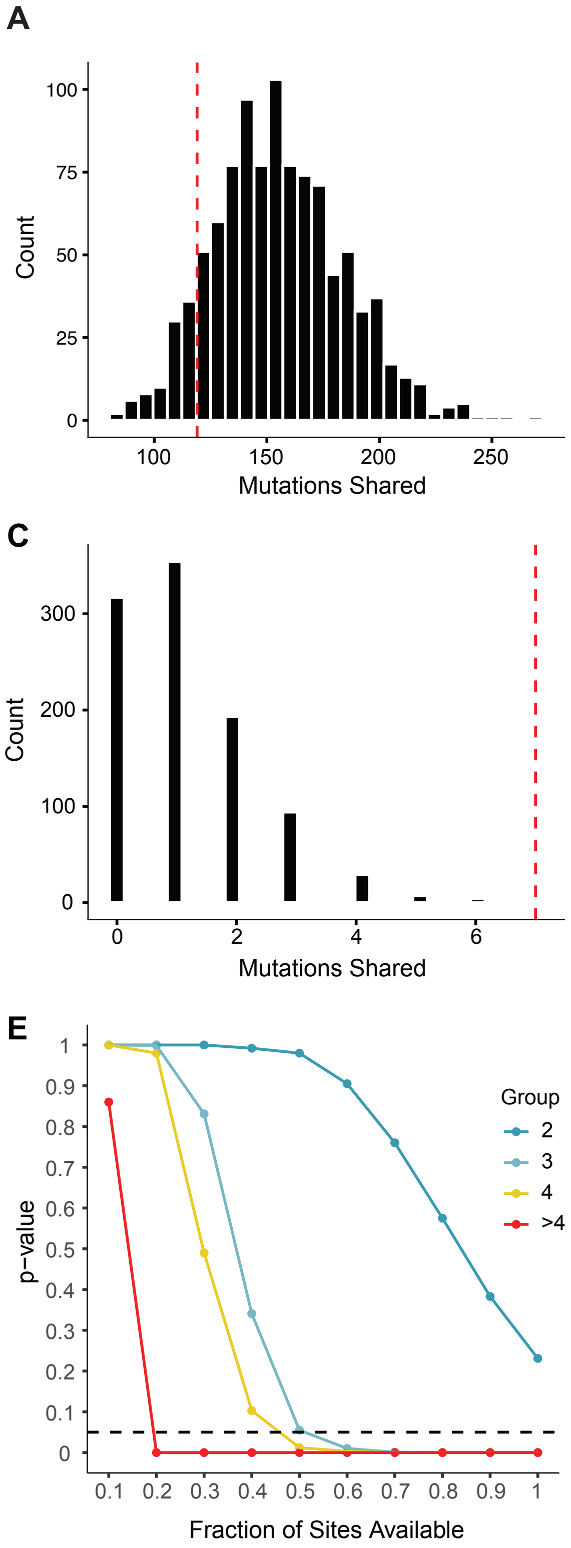

Figure S4. Permutation Tests, Related to Figure 4.

In A - D, the dotted red line is the observed number of mutations shared across a given number of individuals, and the bars show the results of 1000 random permutations. Results are shown for mutations shared across two individuals (A), three individuals (B), four individuals (C), and greater than four individuals (D). P-values are designated as the proportion of random permutations equal or greater than the observed number of shared mutations. In E, the p-values for each group are shown as a function of the genome fraction available for mutations. The horizontal dotted line represents alpha = 0.05.

A

Frequency[1]	0.072	0.135	0.121	0.132	0.152	0.081	0.402	0.097	0.088	0.169	0.064	0.116	0.109	0.126	0.058	0.102	0.13	0.125
**Position[2]**	396	481	888	1035	1510	1593	1641	2006	2115	3184	3352	3579	4143	4207	4665	4692	4707	4716
396	2421	NA	NA	NA	NA	NA	NA	NA	NA	NA	NA	NA	NA	NA	NA	NA	NA	NA
481	1457	2636	NA	NA	NA	NA	NA	NA	NA	NA	NA	NA	NA	NA	NA	NA	NA	NA
888	0	0	1804	NA	NA	NA	NA	NA	NA	NA	NA	NA	NA	NA	NA	NA	NA	NA
1035	0	0	819	2369	NA	NA	NA	NA	NA	NA	NA	NA	NA	NA	NA	NA	NA	NA
1510	0	0	0	0	3199	NA	NA	NA	NA	NA	NA	NA	NA	NA	NA	NA	NA	NA
1593	0	0	0	0	2032	3621	NA	NA	NA	NA	NA	NA	NA	NA	NA	NA	NA	NA
1641	0	0	0	0	1447	2968	3931	NA	NA	NA	NA	NA	NA	NA	NA	NA	NA	NA
2006	0	0	0	0	0	0	0	4383	NA	NA	NA	NA	NA	NA	NA	NA	NA	NA
2115	0	0	0	0	0	0	0	2121	4555	NA	NA	NA	NA	NA	NA	NA	NA	NA
3184	0	0	0	0	0	0	0	0	0	1580	NA	NA	NA	NA	NA	NA	NA	NA
3352	0	0	0	0	0	0	0	0	0	439	6563	NA	NA	NA	NA	NA	NA	NA
3579	0	0	0	0	0	0	0	0	0	0	637	9323	NA	NA	NA	NA	NA	NA
4143	0	0	0	0	0	0	0	0	0	0	0	0	7996	NA	NA	NA	NA	NA
4207	0	0	0	0	0	0	0	0	0	0	0	0	5423	8279	NA	NA	NA	NA
4665	0	0	0	0	0	0	0	0	0	0	0	0	0	0	7860	NA	NA	NA
4692	0	0	0	0	0	0	0	0	0	0	0	0	0	0	7308	8118	NA	NA
4707	0	0	0	0	0	0	0	0	0	0	0	0	0	0	6908	7579	8361	NA
4716	0	0	0	0	0	0	0	0	0	0	0	0	0	0	6405	7089	7664	8326

[1]Frequency of minor variants found in donor of pair 702 at the 19 positions listed.
[2]Positions of 19 minor variants found in donor of pair 702. Table values are the total number of reads overlapping both positions.

B

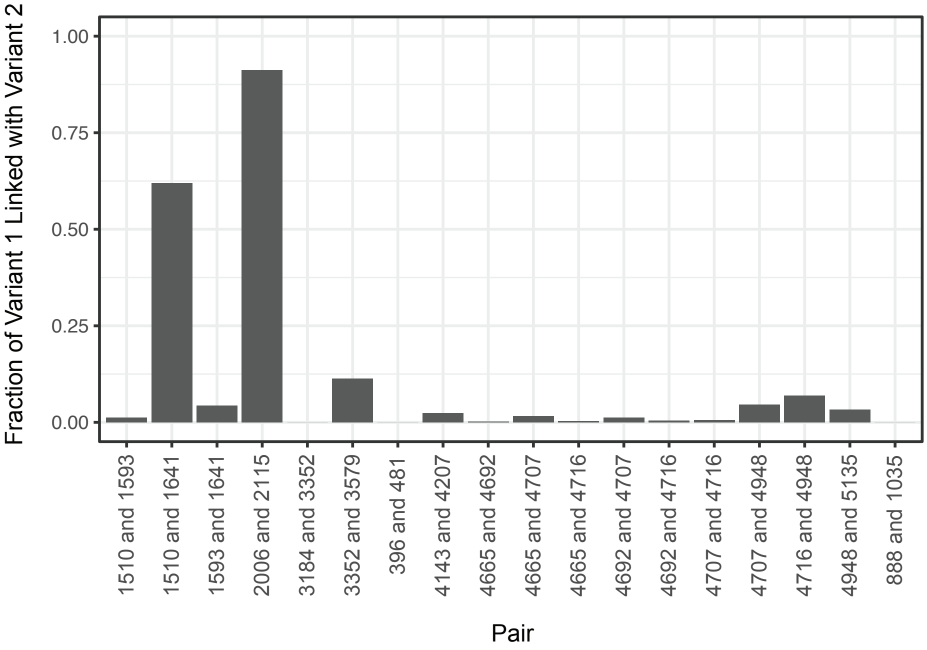

Figure S5. Linkage of Mutations, Related to Figure 5.

(A) The frequency of 20 minor variants present in the donor for pair 702 (top). The table values show the number of sequence reads overlapping each pair of minor variants. (B) Bar chart showing the fraction of minor variant 1 found linked to minor variant 2 in overlapping sequence reads. The 18 pairs of minor variants are shown here by their genome position.
